# Iron Overload Cardiomyopathy in Myelodysplastic Syndrome: A Fatal Outcome After 20 Years of Chronic Transfusions

**DOI:** 10.1016/j.jaccas.2025.104667

**Published:** 2025-08-20

**Authors:** Miyu Hayashida, Shoichiro Nohara, Rei Yamakawa, Aya Nishikido, Yoshihisa Matsushima, Toshiyuki Yanai, Takashi Ishimatsu, Maki Otsuka, Yoshihiro Fukumoto

**Affiliations:** Division of Cardiovascular Medicine, Department of Internal Medicine, Kurume University School of Medicine, Kurume, Japan

**Keywords:** cardiomyopathy, hemochromatosis, iron overload, myelodysplastic syndrome, transfusion-related complications

## Abstract

**Background:**

Iron overload cardiomyopathy (IOC) results from excess iron accumulation in the myocardium, usually because of genetic disorders or secondary hemochromatosis by multiple blood transfusions.

**Case Summary:**

A 42-year-old man with refractory heart failure was referred to our hospital. He had a history of myelodysplastic syndrome, requiring 112 U of blood transfusions over 16 years. Severe left ventricular dysfunction, elevated ferritin levels, and myocardial iron deposition confirmed by endomyocardial biopsy led to the diagnosis of IOC. Despite intensive management, the patient was discharged after death.

**Discussion:**

Early IOC typically starts with asymptomatic diastolic dysfunction. Noninvasive modalities (eg, N-terminal pro–B-type natriuretic peptide, T2-star magnetic resonance imaging) are crucial for early detection. Iron chelation therapy may reverse cardiac dysfunction in the early phase but is less effective in advanced phases, highlighting the need for timely intervention.

**Take-Home Message:**

Early detection, cardiac monitoring, and close collaboration between hematologists and cardiologists are essential to prevent IOC progression.

## Visual Summary

**Figure:**
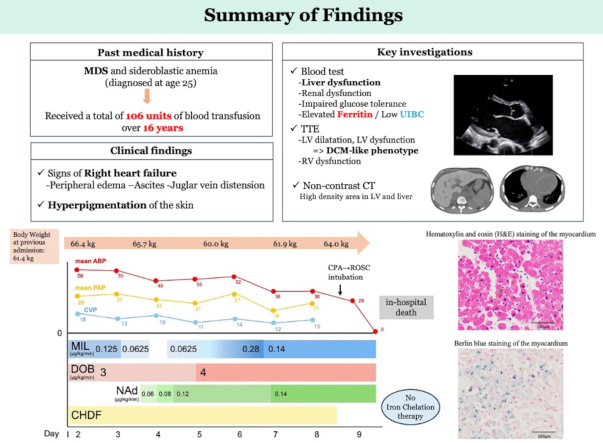

## History of Presentation

A 42-year-old man with refractory heart failure with reduced ejection fraction was referred to our hospital. He initially presented to a previous physician with leg edema and fatigue. The initial diagnosis included chronic anemia and diabetic ketoacidosis, based on impaired glucose tolerance and elevated ketone levels. However, on admission, routine evaluations revealed congestive heart failure with severe left ventricular dysfunction. Despite treatment with diuretics and catecholamines by the cardiologist, his condition deteriorated. He was subsequently referred to our cardiac care unit for further management.

**Take-Home Messages**

• Early detection and regular cardiac monitoring are essential to prevent the progression of IOC in patients with long-term transfusions.
• Timely intervention with iron chelation therapy can improve outcomes, emphasizing the importance of collaboration between hematologists and cardiologists.

On admission, his respiratory rate was 12 breaths/min, and his oxygen saturation was 98% while receiving oxygen at 3 L per minute via nasal cannula. His blood pressure was 96/74 mm Hg under continuous infusion of dobutamine (3 μg/kg/min) and milrinone (0.0625 μg/kg/min). Physical examination revealed jugular vein distension, generalized edema, and abdominal distension because of ascites, consistent with congestive heart failure. Skin examination showed jaundice and hyperpigmentation with a mildly atrophic appearance.

## Past Medical History

The patient was diagnosed with myelodysplastic syndrome (MDS) and sideroblastic anemia at 25 years of age based on bone marrow biopsy findings. An allogeneic stem cell transplant was considered shortly after the initial diagnosis. The patient was registered with the bone marrow donor program at that time. However, the registration was cancelled 2 years later at the patient's request, and he subsequently discontinued outpatient follow-up on his own. Over the subsequent 16 years, he received a cumulative total of 112 U of red blood cell (RBC) transfusions for symptomatic management. Most recently, he underwent a transfusion of 2 U of RBCs 1 month before admission because of fatigue. Before admission, he occasionally experienced dyspnea but was able to perform daily activities without difficulty and continued working in a sales position.

Retrospective information obtained from the referring hospital revealed that cardiomegaly and elevated brain natriuretic peptide levels were noted approximately 11 years before admission, without subsequent cardiac evaluation.

## Differential Diagnosis

Causes of left ventricular dysfunction in the absence of myocardial hypertrophy typically include ischemic cardiomyopathy, sarcoidosis, dilated cardiomyopathy, and other etiologies. Based on the patient's medical history, diabetic cardiomyopathy and secondary hemochromatosis were also considered potential contributing factors to left ventricular dysfunction.

## Investigations

**Figure 1:**
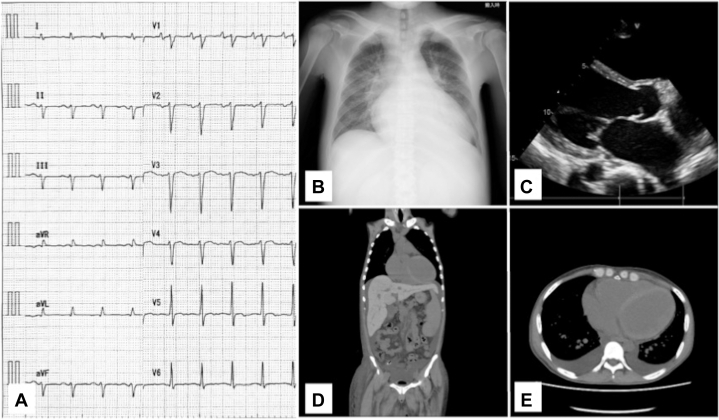
Multimodality Imaging and ECG Findings (A) Electrocardiography at admission showed sinus tachycardia with left axis deviation and no significant ST-T changes. (B) Chest radiograph in the supine position, showed a cardiothoracic ratio of 67%, indicating cardiomegaly and pulmonary congestion. (C) Transthoracic echocardiography revealed dilated left ventricle and reduced ejection fraction. (D) Coronal view of noncontrast computed tomography scan demonstrating high-attenuation areas in the myocardium and liver. (E) Axial view also demonstrates high-attenuation areas, primarily in the left ventricular wall.

The initial blood tests showed elevated liver enzymes, hyperbilirubinemia, hypoalbuminemia, and renal dysfunction. Electrolyte imbalances included hyponatremia and hyperkalemia. Hyperglycemia and a prolonged prothrombin time/international normalized ratio were also noted. N-terminal pro–B-type natriuretic peptide was significantly elevated, and ferritin levels were markedly high. The arterial blood gas analysis showed no major acid-base disturbances (Table 1). Electrocardiography revealed sinus tachycardia with left axis deviation and no significant ST-T changes (Figure 1). Chest radiograph demonstrated a cardiothoracic ratio of 67%, consistent with cardiomegaly, along with pulmonary congestion. Transthoracic echocardiography (TTE) showed diffuse left ventricular hypokinesis with an ejection fraction of 20% and significant left ventricular dilatation (left ventricular end-diastolic dimension: 68 mm, left ventricular end-systolic dimension: 64 mm). Additionally, tricuspid annular plane systolic excursion was 14 mm, suggesting right ventricular dysfunction (Video 1, Video 2, Video 3). Noncontrast computed tomography revealed bilateral pleural effusion, increased left ventricular and hepatic density, hepatosplenomegaly, and ascites (Figure 1).

**Table 1:** Initial Laboratory and Arterial Blood Gas Results Under 2 L/min Nasal Oxygen

Blood test	
AST	146 U/L
ALT	138 U/L
LDH	182 U/L
T.Bil	5 mg/dL
D.Bil	1.5 mg/dL
Alb	1.5 g/dL
UN	49 mg/dL
Creatinine	1.44 mg/dL
Na	124 mmol/L
K	5.4 mmol/L
Cl	90 mmol/L
Glucose	312 mg/dL
HbA1c	8.1 %
NT-proBNP	6137 pg/mL
Ferritin	6578.4 ng/mL
UIBC	<4 μg/dL
WBC	7,500 /μL
RBC	404 × 10^4^/μL
Hb	9.1 g/dL
Hct	29.4 %
Plt	17.4 × 10^3^/μL
APTT	61.6 s
PT-INR	2.53 PT-INR
Arterial blood gas	
pH	7.4
PaO_2_	82.5 mm Hg
PaCO_2_	40.4 mm Hg
HCO3-	25 mmol/L
Lactate	1.5 mmol/L

Alb = albumin; ALT = alanine aminotransferase; APTT = activated partial thromboplastin time; AST = aspartate aminotransferase; Cl = chloride; D.Bil = direct bilirubin; Hb = hemoglobin; HbA1c = hemoglobin A1c; HCO3- = bicarbonate; Hct = hematocrit; K = potassium; LDH = lactate dehydrogenase; Na = sodium; NT-proBNP = N-terminal pro–B-type natriuretic peptide; PaCO_2_ = arterial partial pressure of carbon dioxide; PaO_2_ = arterial partial pressure of oxygen; Plt = platelets; PT-INR = prothrombin time/international normalized ratio; RBC = red blood cells; T.Bil = total bilirubin; UIBC = unsaturated iron-binding capacity; UN = urea nitrogen; WBC = white blood cells.

**Figure 2:**
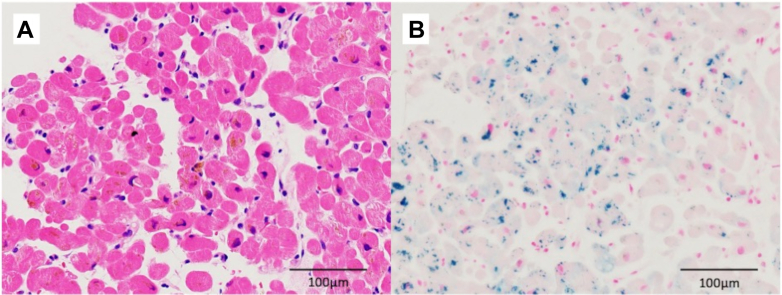
Histopathologic Image of the Myocardium (A) Hematoxylin and eosin staining of the myocardium. (B) Berlin blue staining of the myocardium. The blue areas indicate iron deposition in the myocardium.

Cardiac catheterization was performed for further evaluation of cardiomyopathy. Coronary angiography showed no significant coronary artery stenosis. Hemodynamic assessment via right heart catheterization under continuous infusion of dobutamine (3 μg/kg/min) and milrinone (0.125 μg/kg/min) demonstrated elevated right heart pressures (mean right atrial pressure: 18 mm Hg, mean pulmonary artery pressure: 26 mm Hg, mean pulmonary capillary wedge pressure: 20 mm Hg) and low cardiac output (cardiac index: 2.1 L/min/m^2^, mixed venous oxygen saturation: 49.2%). Endomyocardial biopsy revealed no marked myocyte degeneration or fibrosis. Hematoxylin and eosin staining showed cytoplasmic brown pigment, and Berlin blue staining indicated iron deposition (Figure 2).

## Management

A Swan-Ganz catheter was placed via the right internal jugular vein for hemodynamic assessment and catecholamine titration, including dobutamine and milrinone. Noradrenaline was added to address hypotension and decreased systemic vascular resistance. As the patient's renal failure progressed because of low cardiac output and congestion, diuretic resistance developed, resulting in oliguria despite continuous furosemide infusion at 100 mg/d. Consequently, continuous hemodiafiltration was initiated for volume overload management.

Myocardial biopsy confirmed iron deposition within the myocardium, leading to a diagnosis of iron overload cardiomyopathy (IOC). In this case, the patient had already exhibited severely reduced left ventricular contractility, along with organ damage, including liver dysfunction and diabetes mellitus, secondary to hemochromatosis, suggesting a terminal disease stage. Although iron chelation therapy is the standard treatment for IOC, its efficacy in the chronic phase remains uncertain, and it carries a risk of severe adverse effects, including hepatotoxicity, nephrotoxicity, and agranulocytosis. Given these considerations, iron chelation therapy was not initiated. Therapeutic phlebotomy was not considered because of persistent transfusion-dependent anemia and unstable hemodynamics.

Heart transplantation and left ventricular assist device implantation were considered after multidisciplinary discussions. However, extensive iron deposition in the liver and pancreas, along with dialysis-dependent renal dysfunction, indicated irreversible organ damage. Consequently, these advanced therapies were deemed unsuitable. Additionally, mechanical circulatory support was not pursued because of the absence of a viable long-term treatment strategy.

## Outcome and Follow-Up

Given the chronic nature of the patient's iron overload, recovery from refractory heart failure, systemic congestion, and a low-flow state was deemed unlikely. Because of poor responsiveness to treatment, the focus of care shifted to end-of-life management. The patient died on day 10.

## Discussion

We encountered a case of IOC presenting as refractory heart failure secondary to prolonged blood transfusions over many years. Unfortunately, the patient was in the chronic and irreversible phase of IOC at the time of treatment initiation. As a result, the therapeutic response was poor, and the patient ultimately succumbed to the disease.

IOC results from excessive iron accumulation in the myocardium, which may arise from genetic disorders of iron metabolism or secondary hemochromatosis because of multiple blood transfusions in conditions such as thalassemia, MDS, myelofibrosis, aplastic anemia, and Diamond-Blackfan anemia.^1^ Among patients with MDS, chronic transfusions are common, and approximately one-half of those receiving 75 to 100 U of transfused blood develop clinically significant myocardial iron overload.^2^ In its early phase, IOC typically manifests as diastolic dysfunction and remains asymptomatic for a period.

Kremastinos et al^3^ identified 2 distinct phenotypes of IOC. The first is dilated cardiomyopathy, characterized by left ventricular dilation and impaired systolic function, leading to progressive myocardial damage and reduced cardiac output. The second is restrictive cardiomyopathy, defined by impaired ventricular filling, pulmonary hypertension, and right-sided heart failure. This phenotype is associated with advanced myocardial fibrosis and severe iron deposition in cardiac tissue, resulting in diastolic dysfunction. This case was classified as the dilated cardiomyopathy type because of significant left ventricular dilatation and impaired systolic function. However, because no previous TTE records were available, the timeline of systolic dysfunction progression remains unknown. Immune and inflammatory mechanisms are thought to contribute to the pathogenesis of IOC. The precise duration of iron exposure or the cumulative transfusion volume required to induce cardiomyopathy also remains unclear because these factors vary based on individual iron metabolism.

Noninvasive modalities (eg serum ferritin, TTE, magnetic resonance imaging) are valuable for cardiac monitoring. In Japan, national guidelines recommend monitoring serum ferritin every 3 months and performing magnetic resonance imaging as needed in patients with a history of receiving ≥40 U of RBC transfusions.^4^ The patient underwent frequent ferritin testing and had already exceeded 3,000 ng/mL 10 years before admission, but no intervention was initiated and he remained under observation.

Early diastolic dysfunction can be detected using serial Doppler echocardiography; however, it may remain subclinical.^5^ N-terminal pro–B-type natriuretic peptide levels have been reported to rise before Doppler abnormalities appear in patients with β- thalassemia.^6^ Combined monitoring of N-terminal pro–B-type natriuretic peptide and TTE may facilitate early detection of cardiac iron overload. T2-star magnetic resonance imaging is a reliable method for the early detection and quantification of myocardial iron overload. Anderson et al^7^ demonstrated that cardiovascular T2-star magnetic resonance imaging provides an accurate assessment of myocardial iron levels, offering significant advantages over traditional diagnostic methods in patients requiring long-term transfusions. In this case, magnetic resonance imaging was considered but not performed because of hemodynamic instability and ongoing continuous hemodiafiltration.

Iron chelation therapy in the early phase may reverse cardiac dysfunction, but its efficacy in the chronic phase remains uncertain. It is recommended for patients requiring long-term blood transfusions, including those with β-thalassemia, sickle cell disease, and aplastic anemia.^8^ However, the adverse effects—such as nephrotoxicity, hepatotoxicity, acute respiratory distress syndrome, and gastrointestinal toxicity—can be significant, especially in patients with poor general condition, as in this case. In patients with β-thalassemia major and heart failure, prognosis is often <1 year, highlighting the need for early intervention to improve clinical outcomes.

Early intervention requires a collaborative approach between hematologists and cardiologists. A structured protocol including echo surveillance strategies can facilitate timely initiation of iron chelation therapy and other appropriate measures, potentially preventing the progression of heart failure.

## Conclusions

This case illustrates the severe consequences of chronic, advanced-phase IOC resulting from long-term blood transfusions. Early detection and timely initiation of iron chelation therapy are critical in preventing the progression of IOC. This case emphasizes the importance of regular cardiac monitoring and proactive management strategies to mitigate cardiac complications in patients requiring long-term transfusion therapy.

**Visual Summary:**
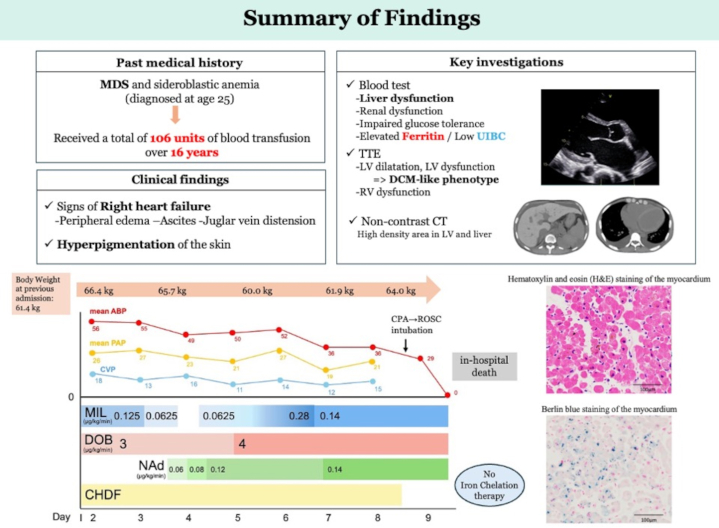
Iron Overload Cardiomyopathy Due to Chronic Transfusion

## Funding Support and Author Disclosures

The authors have reported that they have no relationships relevant to the contents of this paper to disclose.

## Appendix

Video 1 Transthoracic Echocardiography in the Parasternal Long-Axis View Showing Severely Reduced Left Ventricular Systolic Fuction

Video 2 Transthoracic Echocardiography in the Parasternal Short-Axis View Demonstrating Reduced Left Ventricular Contractility

Video 3 Apical 4-Chamber Transthoracic Echocardiographic View Showing Marked Biventricular Dysfunction and a Dilated Left Ventricle1
